# TNF-alpha inhibitors biosimilar use in France: a nationwide population-based study using the French National Health Data System

**DOI:** 10.1038/s41598-022-24050-7

**Published:** 2022-11-15

**Authors:** Hugo Jourdain, Léa Hoisnard, Emilie Sbidian, Mahmoud Zureik

**Affiliations:** 1 EPI-PHARE, French National Agency for Medicines and Health Products Safety (ANSM) and French National Health Insurance (CNAM), 143-147 Boulevard Anatole France, 93285 Saint-Denis, France; 2 Fédération Hospitalo-Universitaire TRUE InnovaTive theRapy for immUne disordErs, Assistance Publique-Hôpitaux de Paris (AP-HP), Henri Mondor Hospital, 94010 Créteil, France; 3 INSERM, Centre d’Investigation Clinique 1430, 94010 Créteil, France; 4 EpiDermE Epidemiology in Dermatology and Evaluation of Therapeutics, EA7379, Paris Est Créteil University UPEC, F-94010 Créteil, France; 5 Department of Dermatology, Hôpital Henri Mondor, 51 Avenue du Maréchal de Lattre de Tassigny, 94000 Créteil, France; 6 University Paris-Saclay, UVSQ, University Paris-Sud, Inserm, Anti-Infective Evasion and Pharmacoepidemiology, CESP, Montigny le Bretonneux, France

**Keywords:** Epidemiology, Biological therapy, Rheumatoid arthritis, Crohn's disease, Ulcerative colitis, Skin diseases, Uveal diseases, Spondyloarthritis, Ankylosing spondylitis, Psoriatic arthritis, Inflammatory bowel disease

## Abstract

TNF-alpha inhibitors have revolutionized the therapeutic care in chronic inflammatory diseases. Several biosimilar products were commercialized at their patent expiry, substantially decreasing the cost of treatment. This longitudinal descriptive study aimed at assessing infliximab, etanercept and adalimumab biosimilar penetration rates using data of the French National Health Data System. A total of 207,118 new or prevalent users from the date of first biosimilar commercialization in France (respectively January 2015, May 2016 and October 2018) were included in the study and followed until September 30, 2021. Biosimilars represented respectively 78%, 46% and 53% of the overall initiations, and 94%, 66% and 60% last year’s initiations. A total of 46%, 19% and 17% of originator product prevalent users switched for a biosimilar during the follow-up. Biosimilar penetration rate was much higher for infliximab than for its counterparts, due to its hospital delivery modality. Biosimilar initiation and originator-to-biosimilar switch tended to be observed more in rheumatology than in the other specialties. Biosimilar use was mostly consistent across patient socio-demographic characteristics. Biosimilar initiation rate increased rapidly from their market arrival and originator-to-biosimilar switch rate remained moderate, highlighting the need and usefulness of political action and biosimilar use tracking.

## Introduction

Tumor necrosis factor alpha (TNF-alpha) inhibitors are a class of biotherapies that has revolutionized the therapeutic care in chronic inflammatory diseases, such as psoriasis, rheumatoid arthritis and Crohn’s disease^1^. Infliximab, etanercept and adalimumab were marketed respectively in 2000, 2003 and 2005 at expensive costs, leading to an issue of either access for patients or healthcare system sustainability due to the large number of patients treated. TNF-alpha inhibitors patent expiry and biosimilars progressive market approvals partly answered these issues by lowering the cost of treatment^2^. Biosimilars are highly similar formulations of the originator product (same dosage forms and volumes), approved through a rigorous regulatory process including head-to-head clinical trials to demonstrate their bioequivalence, i.e. efficacy and safety^3–5^.

Biosimilar use is highly dependent of the internal policy in each country. Biosimilars have been more implanted in Europe than in the United States^6^ or Japan^7^, thanks to accelerated market approvals and country level policies: fast reimbursement, financial incentives to push biosimilar prescription, authorization of switching or substitution of the originator product by the biosimilar at pharmacy level^8,9^. Despite some utilization studies^10,11^, data on the level of use of biosimilars in Europe, in particular TNF-alpha inhibitors, remain scarce.

In France, as TNF-alpha inhibitors are fully reimbursed and widely used, biosimilar utilization has been a particularly important challenge for the healthcare system sustainability: to date, three TNF-alpha inhibitors have been biosimilarized, infliximab in 2015, etanercept in 2016 and adalimumab in 2018^12^. It is worth noting that TNF-alpha inhibitors initial prescription and annual renewal are done at the hospital level, along with delivery for infliximab but etanercept and adalimumab delivery is carried out by retail pharmacists. A recent study^13^ based on French data, but limited to hospital dispensations, showed that TNF-alpha inhibitor biosimilar penetration rate was close to 80%.

The aim of the present descriptive study was to assess the level of biosimilar use in France of these three TNF-alpha inhibitors with a marketed biosimilar, in order to understand the key drivers for biosimilar prescription and be able to improve their penetration, with a national payer perspective. For each molecule, we estimated penetration rate globally, and penetration rate depending on the pathology treated, the specialty or patient characteristics. We focused our study on two populations: initiators within the period of biosimilar commercialization, to compare biosimilar versus originator initiators, and prevalent users at the date of first biosimilar commercialization, to study their switches along time. We used the French National Health Data System (Système National des Données de Santé, SNDS), the French comprehensive health claims database, including inpatient and outpatient reimbursements, to address this issue.

## Results

### Overall population

**Figure 1:**
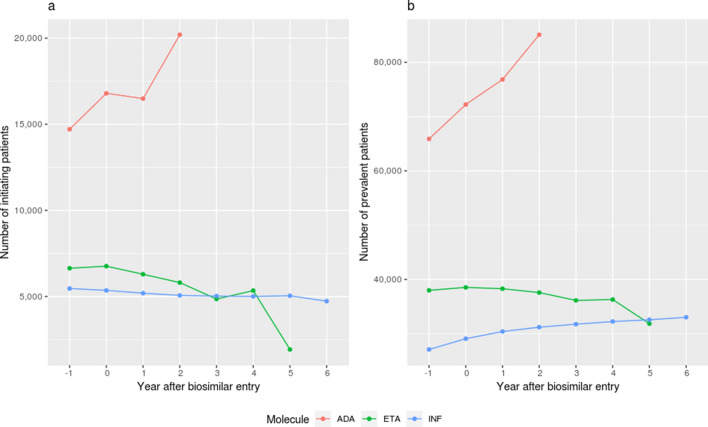
Number of patients initiating TNF-alpha inhibitor (**a**) and prevalent users of TNF-alpha inhibitor (**b**) between year − 1 and 6 (if applicable) after first biosimilar entry for each molecule. ADA, ETA and INF refer to adalimumab, etanercept and infliximab respectively. Adalimumab year 2 was 98% complete, infliximab year 6 67% and etanercept year 5 39%, from market entry date anniversary to study end date.

During the study period, 115,656 unique patients initiated a TNF-alpha inhibitor treatment, for a total of 217,756 unique prevalent users. Adalimumab utilization increased while it remained stable for infliximab and etanercept. Adalimumab initiations increased from 14,711 in year − 1 to 20,198 in year 2, totalizing 85,082 users in year 2, whereas etanercept initiations went from 6639 in year − 1 down to 5341 initiators in year 4, with 36,306 users in year 4, and infliximab from 5460 in year − 1 to 5041 in year 5, with 32,577 users in year 5. (Fig. 1).

**Figure 2:**
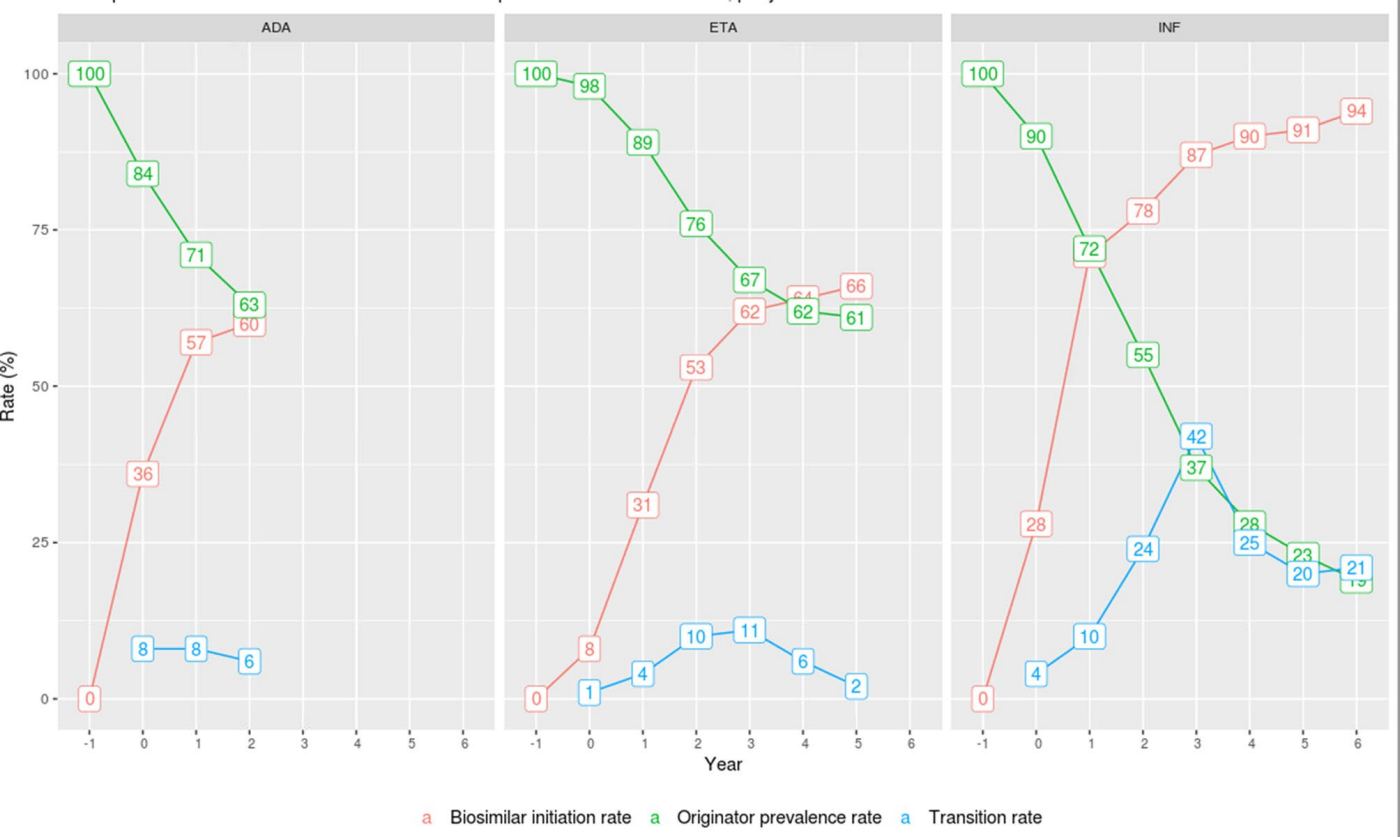
TNF-alpha inhibitors’ biosimilar initiation rate, originator prevalence rate and transition rate between year − 1 and 6 (if applicable) after first biosimilar entry for each molecule. ADA, ETA and INF refer to adalimumab, etanercept and infliximab respectively.

Overall, biosimilar initiations increased from their market entry to the end of the study. Infliximab biosimilar initiation rate increased faster than etanercept and adalimumab, with already 78% of initiations by biosimilars at year 2 versus 60% for adalimumab and 53% for etanercept. With respective follow-ups of 7, 6 and 3 years on their market entry, biosimilar initiation rates reached 94% for infliximab, 66% for etanercept and 60% for adalimumab. On the other hand, transition rate was much higher in infliximab (42% at peak in year 3) versus etanercept (11% at peak in year 3) or adalimumab (8% at peak in year 0). As a matter of fact, in the last year of follow-up, originator product infliximab represented only 19% of the total infliximab use; it was still much higher for etanercept (61%) and adalimumab (63%). (Fig. 2).

**Figure 3:**
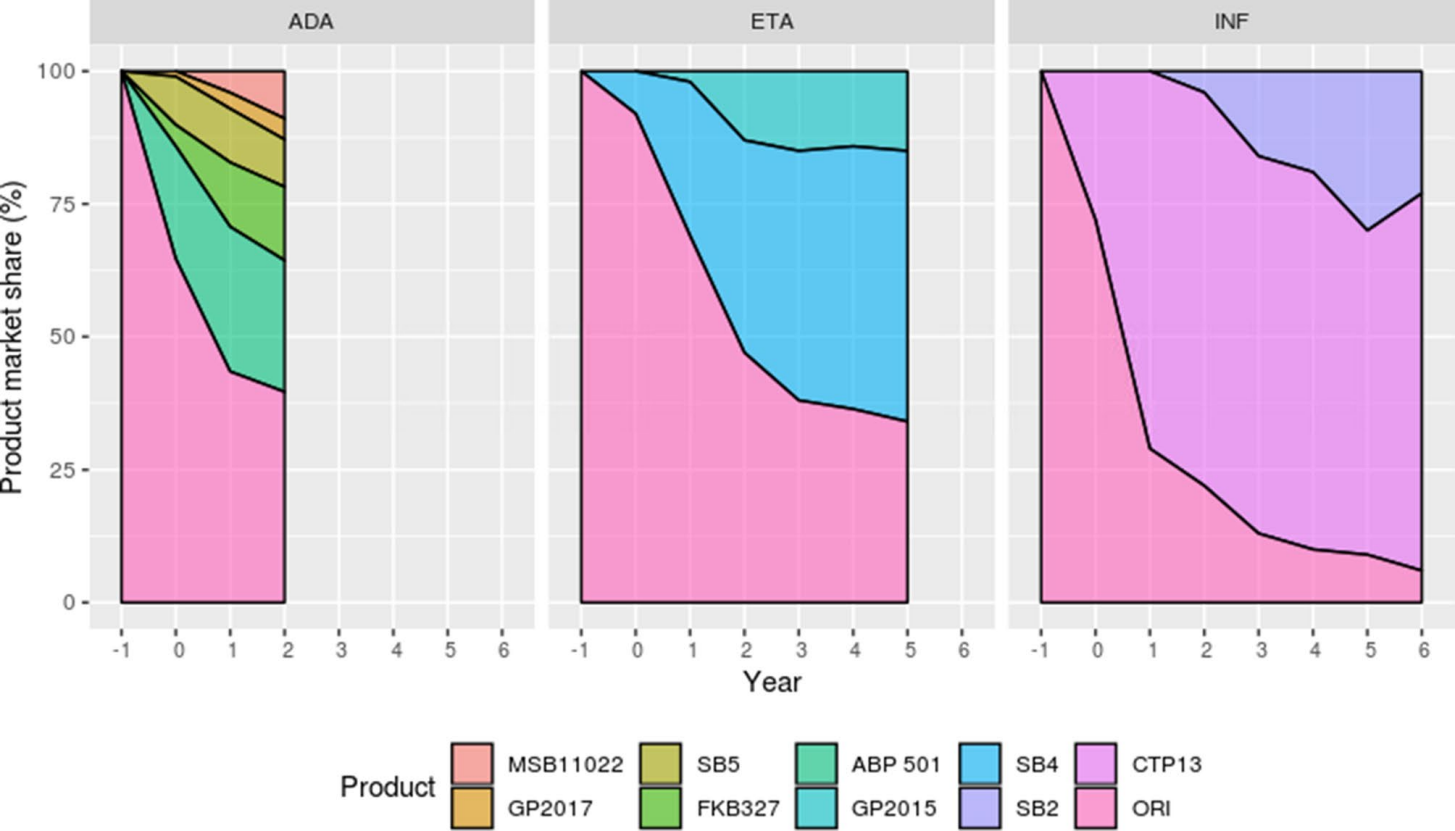
TNF-alpha inhibitors initiation rate by product between year − 1 and 6 (if applicable) after first biosimilar entry for each molecule. ADA, ETA and INF refer to adalimumab, etanercept and infliximab respectively. ORI refers to originator product.

Regarding biosimilar initiation rate, the first biosimilar product to come to market (CTP13, SB4, ABP501) reached the highest penetration rate versus its competitors (respectively representing, for the last year of follow-up, 73%, 51%, 25% of the infliximab, etanercept and adalimumab initiations) (Fig. 3).

### Patient characteristics at inclusion

In the initiation population (i), we included 25,950 infliximab patients (78% of biosimilar initiators), 27,727 etanercept patients (46% of biosimilar initiators) and 48,768 adalimumab patients (53% of biosimilar initiators), for a total of 102,445 initiators. Patient characteristics were mostly similar between originator and biosimilar initiators (Table 1 and Supplementary Table S1).

**Table 1:** Initiators characteristics at inclusion and during the study follow-up, by molecule and product type.

	Infliximab		Etanercept		Adalimumab	
	Biosimilar	Originator	Biosimilar	Originator	Biosimilar	Originator
**Effective**	20,208 (78)	5742 (22)	12,713 (46)	15,014 (54)	26,033 (53)	22,735 (47)
**Female**	10,377 (51)	3012 (52)	7907 (62)	9529 (63)	14,379 (55)	12,599 (55)
**Age at inclusion (years)**	–	–	–	–	–	–
Mean (SD)	43.9 (16.3)	43.9 (16.1)	51.9 (15.2)	50.7 (15.1)	45.2 (15)	43.6 (15.4)
18–29	4666 (23)	1315 (23)	1004 (8)	1298 (9)	4576 (18)	4862 (21)
30–39	4285 (21)	1188 (21)	1992 (16)	2531 (17)	5555 (21)	5063 (22)
40–49	3920 (19)	1185 (21)	2466 (19)	3311 (22)	5632 (22)	4828 (21)
50–59	3464 (17)	982 (17)	3150 (25)	3577 (24)	5412 (21)	4110 (18)
60–69	2404 (12)	671 (12)	2328 (18)	2531 (17)	3231 (12)	2516 (11)
70 +	1469 (7)	401 (7)	1773 (14)	1766 (12)	1627 (6)	1356 (6)
**Pathology**	–	–	–	–	–	–
**Gastro-enterology**	–	–	–	–	–	–
Crohn's Disease	7914 (39)	2407 (42)	0 (0)	0 (0)	5583 (21)	7127 (31)
Ulcerative colitis	4358 (22)	1058 (18)	0 (0)	0 (0)	2697 (10)	4122 (18)
**Rheumatology**	–	–	–	–	–	–
Rheumatoid arthritis	1607 (8)	446 (8)	5997 (47)	5618 (37)	3888 (15)	1717 (8)
Ankylosing spondylitis	3358 (17)	781 (14)	4441 (35)	5356 (36)	8449 (32)	3811 (17)
Psoriatic arthritis	448 (2)	142 (2)	688 (5)	920 (6)	1387 (5)	664 (3)
**Dermatology**	–	–	–	–	–	–
Psoriasis	862 (4)	294 (5)	573 (5)	1320 (9)	1926 (7)	2013 (9)
Hidradenitis Suppurativa	0 (0)	0 (0)	0 (0)	0 (0)	52 (0)	267 (1)
**Ophtalmology**	–	–	–	–	–	–
Uveitis	0 (0)	0 (0)	0 (0)	0 (0)	204 (1)	538 (2)
**Undetermined**	1661 (8)	614 (11)	1014 (8)	1800 (12)	1847 (7)	2476 (11)
**Follow-up duration, mean (SD)**	1.6 (1.5)	2.4 (2.1)	1.3 (1.1)	1.7 (1.6)	0.9 (0.7)	1.1 (0.9)
**Follow-up duration, median (IQR)**	1 (0.5–2.3)	1.5 (0.6–4)	0.8 (0.4–2)	1 (0.4–2.8)	0.7 (0.4–1.3)	0.8 (0.4–1.8)
**Number of dispensings, mean (SD)**	11.7 (10.9)	16.4 (15.2)	13.8 (12.8)	17 (17.4)	10.4 (8.5)	12.5 (10.5)
**Discontinuation, molecule switch or death**	10,070 (50)	3830 (67)	6228 (49)	9763 (65)	8967 (34)	9338 (41)
**Transitions**	816 (4)	2031 (35)	225 (2)	1575 (10)	370 (1)	1396 (6)
Time to switch, median (IQR)	0.9 (0.4–1.7)	1.6 (0.6–2.6)	0.9 (0.4–1.7)	1.6 (0.7–2.5)	0.5 (0.3–0.9)	0.6 (0.2–1.1)
**Retransitions**	1226 (6)	439 (8)	712 (6)	528 (4)	1634 (6)	417 (2)
Time to switch, median (IQR)	0.5 (0.2–1.2)	2 (1–3.2)	0.4 (0.2–0.9)	2.2 (1.3–3.1)	0.3 (0.1–0.6)	0.8 (0.4–1.5)
**Biotransitions**	2277 (11)	300 (5)	291 (2)	47 (0)	644 (2)	71 (0)
Time to switch, median (IQR)	1.2 (0.6–2.2)	3.2 (2.4–4.6)	0.5 (0.2–1.1)	2.7 (1.4–3.5)	0.4 (0.2–0.8)	1.2 (0.7–1.7)

Figures are no. (%) unless stated otherwise.

IQR, interquartile range; SD, standard deviation.

Infliximab new users were mostly women (52%), aged 44 years on average and with a uniform repartition of quintiles of deprivation index. Infliximab new patients were mostly treated for inflammatory bowel diseases (40% in Crohn’s disease and 21% in ulcerative colitis), then for rheumatology pathologies (16% in ankylosing spondylitis, 8% in rheumatoid arthritis, 2% in psoriatic arthritis) and then for psoriasis (4%).

Etanercept new users were mostly women (63%), aged 51 years on average. Etanercept new patients were mostly treated for rheumatology pathologies (35% in ankylosing spondylitis, 42% in rheumatoid arthritis, 6% in rheumatoid psoriasis) and then for psoriasis (7%).

Adalimumab new users were mostly women (55%), aged 44 years on average. Adalimumab new patients were mostly treated for inflammatory bowel diseases (26% in Crohn’s disease and 14% in ulcerative colitis), and for rheumatology pathologies (25% in ankylosing spondylitis, 11% in rheumatoid arthritis, 4% in rheumatoid psoriasis) and then for dermatology (8% for psoriasis and 1% for hidradenitis suppurativa) and uveitis (2%).

Corticosteroid use before initiation was consistent within molecules, with 69% of patients having used at least once corticosteroids. Respectively 37%, 69% and 50% of infliximab, etanercept and adalimumab new users had at least one delivery of a NSAID before initiation. Non-biological systemic drugs and biological and targeted drugs had been used respectively by 52–54% and 11–17% of TNF-alpha inhibitor initiators. Comorbidities were evenly distributed between biosimilar and originator products and across molecules.

**Figure 4:**
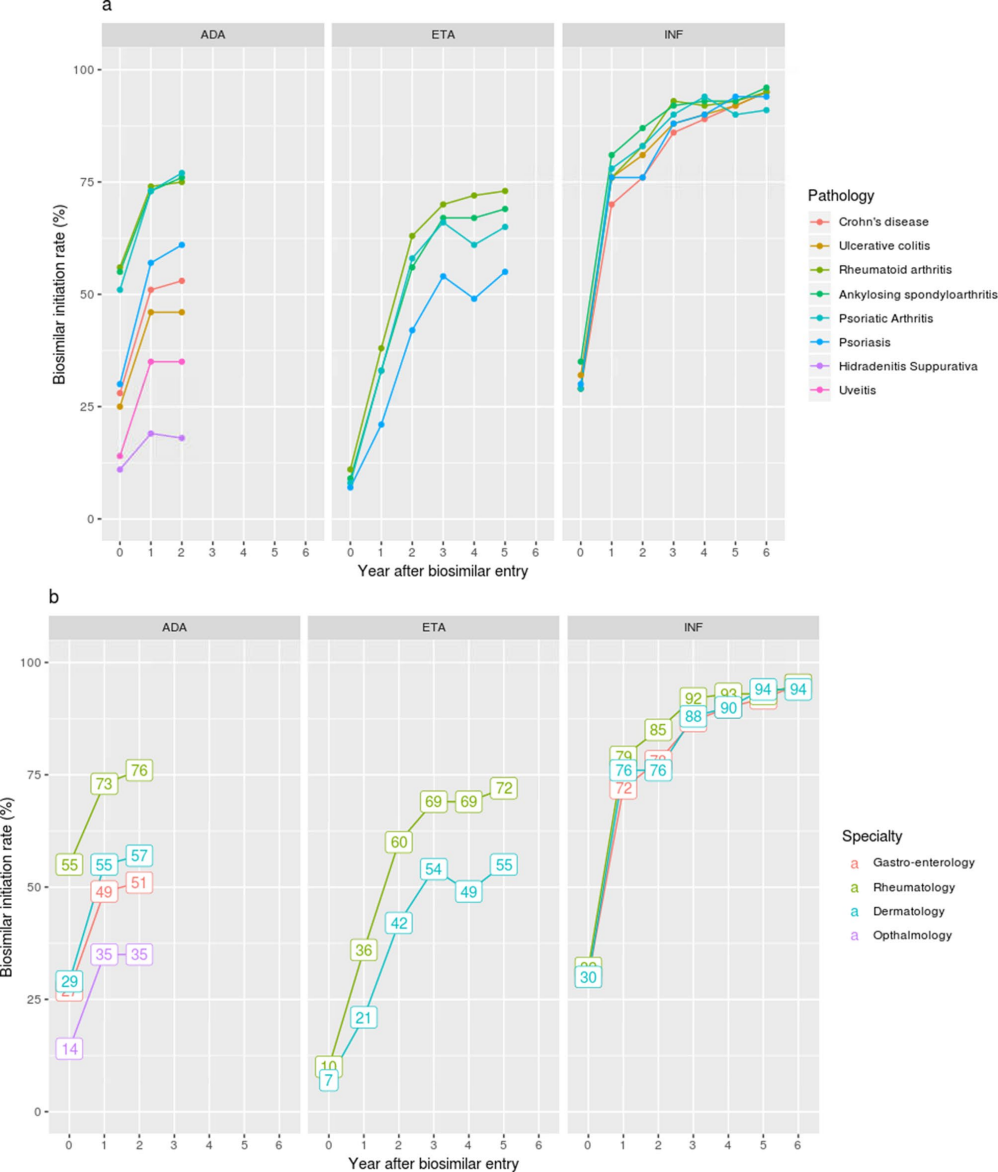
TNF-alpha inhibitors’ biosimilar initiation rate by pathology (**a**) and specialty (**b**) between year 0 and 6 (if applicable) after first biosimilar entry for each molecule. ADA, ETA and INF refer to adalimumab, etanercept and infliximab respectively.

The pathology treated and thus the specialty of interest (after grouping pathologies) had an important impact on biosimilar versus originator initiation. Except infliximab, for which biosimilar initiation rates were almost uniform whatever the specialty, we observed a gradient of biosimilar use according to the pathology: biosimilars were mostly used in rheumatology, then in dermatology, then in gastro-enterology and finally ophthalmology. (Fig. 4).

The prevalent population of TNF-alpha inhibitors users at study start date (ii) was constituted of 20,254 infliximab patients, 31,038 etanercept patients and 53,381 adalimumab patients, for a total of 104,673 prevalent users. (Table 2 and Supplementary Table S2).

**Table 2:** Prevalent users characteristics at inclusion and during the study follow-up, by molecule.

	**Infliximab**	**Etanercept**	**Adalimumab**
**Effective**	20,254	31,038	53,381
**Female**	10,306 (51)	17,363 (56)	26,738 (50)
**Age at inclusion (years)**	–	–	–
Mean (SD)	45 (15.3)	53.3 (14.5)	46.7 (15.2)
18–29	3753 (19)	1716 (6)	8039 (15)
30–39	4149 (20)	3984 (13)	10,654 (20)
40–49	4634 (23)	6665 (21)	11,995 (22)
50–59	3874 (19)	7771 (25)	11,120 (21)
60–69	2569 (13)	6725 (22)	7459 (14)
70 +	1275 (6)	4177 (13)	4114 (8)
**Pathology**	–	–	–
**Gastro-enterology**	–	–	–
Crohn's Disease	8507 (42)	0 (0)	17,221 (32)
Ulcerative colitis	2784 (14)	0 (0)	5008 (9)
**Rheumatology**	–	–	–
Rheumatoid arthritis	2032 (10)	12,822 (41)	7366 (14)
Ankylosing spondylitis	4825 (24)	11,191 (36)	13,247 (25)
Psoriatic arthritis	733 (4)	2258 (7)	2433 (5)
**Dermatology**	–	–	–
Psoriasis	1076 (5)	3282 (11)	5084 (10)
Hidradenitis Suppurativa	0 (0)	0 (0)	210 (0)
**Ophtalmology**	–	–	–
Uveitis	0 (0)	0 (0)	536 (1)
**Undetermined**	297 (1)	1485 (5)	2276 (4)
**Follow-up duration, mean (SD)**	4.3 (2.6)	3.6 (2.1)	2.3 (1)
**Follow-up duration, median (IQR)**	5.3 (1.6–6.7)	5.1 (1.5–5.4)	3 (1.6–3)
**Number of dispensings, mean (SD)**	27.7 (18.5)	32.9 (22.6)	23.6 (13.4)
**Discontinuation, molecule switch or death**	11,506 (57)	16,533 (53)	20,449 (38)
**Transitions**	9417 (46)	5907 (19)	9285 (17)
Time to switch, median (IQR)	3.1 (2.3–3.7)	2.9 (2.2–3.6)	1.1 (0.7–1.7)
**Retransitions**	2054 (10)	2183 (7)	3055 (6)
Time to switch, median (IQR)	3.5 (2.4–4.7)	3.5 (2.7–4.2)	1.5 (1.1–2.1)
**Biotransitions**	1895 (9)	161 (1)	541 (1)
Time to switch, median (IQR)	5 (3.3–5.5)	3.7 (3.1–4.6)	1.7 (1.2–2.3)

Figures are no. (%) unless stated otherwise.

IQR, interquartile range; SD, standard deviation.

51%, 56% and 50% of infliximab, etanercept and adalimumab prevalent users were women. Mean age was 45, 53 and 47 years in the respective groups. Pathology repartition was similar to the initiator group.

### Follow-up characteristics and patient switch pathway

Follow-up characteristics and median times are presented in Table 1 for initiators and Table 2 for prevalent users.

During the follow-up period, 35% (n = 2031) of infliximab originator initiators switched to an infliximab biosimilar within 1.6 years after initiation and among them a fifth (n = 439) retransitioned to the originator product less than 6 months later. On the other hand, 6% (n = 1226) of infliximab biosimilar initiators retransitioned 6 months after initiation, and 11% (n = 2277) of biosimilar initiators biotransitioned at least once 1.2 years after initiation.

There were less switches in etanercept and adalimumab initiating patients: transitions occurred respectively in 10% (n = 1575) and 6% (n = 1396) of patients 1.6 and 0.6 years after initiation, with almost a third (n = 528 and n = 417) of them switching back to the originator product less than 6 months later; 6% (n = 712 and n = 1634) of biosimilar initiators retransitioned 0.4 and 0.3 years after initiation and 2% (n = 291 and n = 644) biotransitioned 0.5 and 0.4 years after initiation respectively.

There were more switches in the group of TNF-alpha inhibitors prevalent users. A total of 46% of infliximab prevalent patients (n = 9417) transitioned to a biosimilar 3.1 years after index date, and 22% of them (n = 2054) went back to the originator less than 6 months after, whereas 19% (n = 1895) biotransitioned around 2 years after the transition. Among etanercept prevalent users, 19% of patients (n = 5907) transitioned to a biosimilar 3 years after index date, and 2183 switched back to the originator 0.6 years after, whereas only 161 biotransitioned. Among adalimumab prevalent users, 17% of patients (n = 9285) transitioned to a biosimilar a year after index date, and a third (n = 3055) switched back to the originator less than 6 months after, and 541 biotransitioned.

**Figure 5:**
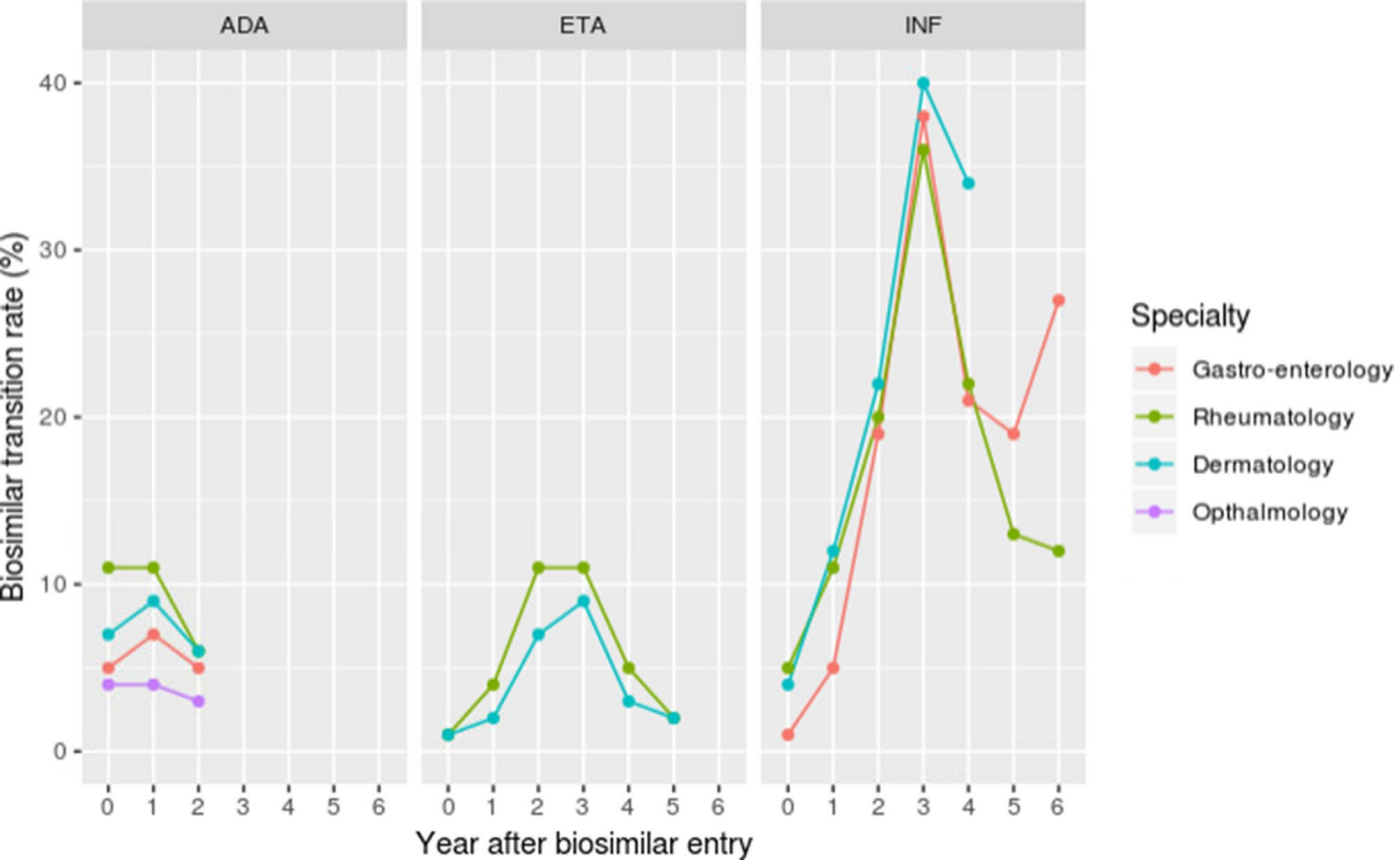
TNF-alpha inhibitors’ biosimilar transition rate by specialty between year 0 and 6 (if applicable) after first biosimilar entry for each molecule. ADA, ETA and INF refer to adalimumab, etanercept and infliximab respectively.

Biosimilar transition rate in prevalent users followed the same scheme in terms of specialty as for biosimilar initiators: low impact of specialty in infliximab prevalent users, and a trend for higher transition rates in rheumatology for etanercept and adalimumab (Fig. 5).

Cumulated major switches (groups of patients reaching more than 500 individuals) are represented in the interactive Sankey diagrams in Supplementary Materials, showing for each group of patients included (initiators and prevalent users) and for each molecule the switch pathways from inclusion to end of study. Patients’ characteristics according to the switch pattern are presented in Supplementary Tables S3–S5, with no major difference between the groups of individuals.

## Discussion

In this large descriptive study, including 102,445 initiating patients from biosimilar market entry and 104,673 prevalent patients at biosimilar market entry, we show that the level of penetration of TNF-alpha inhibitors’ biosimilars varies greatly according to the molecule and the pathology treated. Intrinsic characteristics of patients such as sex, age, deprivation, previous treatments, do not seem to influence biosimilar use.

One could have hypothesized that later biosimilar market entries would benefit from gained experience from the previous entries, with faster biosimilar penetration rates for adalimumab than for etanercept and infliximab. In fact, infliximab biosimilar initiation (78% over the whole period at stake, 94% during the last year of follow-up) and transition (35% in initiators and 46% in prevalent users) rates were high, and increased faster than the others. As a matter of fact, as infliximab is a perfusion medicine and thus exclusively delivered at the hospital level, hospital tendering processes favor biosimilar procurement, leading to a high biosimilar penetration for this specific molecule. On the other hand, etanercept biosimilar penetration rate (46% of total initiations, 66% during the last year of follow-up, and 10–19% of transitions) and adalimumab biosimilar penetration rate (53% of total initiations, 60% during the last year of follow-up, and 6–17% of transitions) are lower. Retail delivery modality indeed allows more originator use. Lower biosimilar use in dermatology and gastro-enterology could also explain this lower penetration rate. Nevertheless, adalimumab biosimilar market entry seems to have benefited from etanercept experience, with a faster biosimilar adhesion. Adalimumab faster biosimilar penetration rate might as well be explained by a more important number of biosimilar marketed products than etanercept. Meanwhile, several measures were taken to improve biosimilar penetration rate in France: the French agency for medicine safety (ANSM) has recommended biosimilar prescription and originator switching since 2016 and hospital-targeting experimentations of financial incentives promoting etanercept and adalimumab biosimilar prescription have been conducted from 2018. Finally, as a result, originator product still represents 61% of etanercept users and 63% of adalimumab users while it represents only 19% of infliximab users.

We report in this study that retransition is not an isolated fact, with respectively 22%, 37% and 32% of infliximab, etanercept and adalimumab prevalent users retransitioning during the study follow-up. This is not consistent with a systematic review^14^ that calculated lower retransition rates, with an average of 8% of retransitions among patients having previously transitioned from originator to biosimilar product a year after the first transition. Nocebo effect has already been suggested to explain biosimilar discontinuation^15,16^, while previous studies did not show any adverse events profiles in transitioning patients compared to non-transitioning patients^17–19^. Additionally, biotherapies (biosimilars and originators) might be subject to manufacturing drift over time^20,21^, ending up with a substantial biological difference between the approved form and the current form and effectiveness differences between products. In our study, the longitudinal visualization of the switch patterns shows a significant number of patients alternating between originator and biosimilar products, suggesting potential switches due to product availability, and thus non-medical isolated switches, or perceived total exchangeability between products.

The main strength of this descriptive study is the large and comprehensive (at the French level) sample provided by the SNDS claims database: we were able to include every initiating and prevalent TNF-alpha inhibitor user within the period of biosimilar products reimbursement, and describe the population.

This study has nevertheless some limitations. Due to the medico-administrative nature of the SNDS, we had to use algorithms and proxies in particular to assess the patient pathologies. However, apart from the choice between multiple pathologies, the medical background codes used in this study relied on ICD-10 and were described and used in previous studies. Besides, we could not have access to clinical or behavioral data that could explain the reasons of use of the originator product rather than the biosimilar. Moreover, these results are specific to the case of France and, considering the fact that internal country policies on biosimilars are a major driver of their penetration, these findings cannot be extrapolated to other countries.

In conclusion, there is still some way to go to implement biosimilar use in the treatment by TNF-alpha inhibitors. Biosimilar prescription should be particularly encouraged at the retail level, especially for dermatologists and gastro-enterologists. The number of patients under the originator product is still very important and is unlikely to decrease rapidly as we observed a relatively low transition rate in etanercept and adalimumab users. Biosimilar initiation rate needs to be tracked further, with specific methods for estimating the impact of public policies, in order to assess the efficacy of country-level policies such as financial incentives for biosimilar prescription or the potential opportunity for pharmacists to substitute the originator product with a biosimilar.

## Methods

### Data source

The SNDS covers almost the totality (> 99%) of the French population—68 million residents. Each person is identified by a unique and anonymous number. The SNDS records comprehensive outpatient (reimbursed drugs and procedures) and inpatient (expensive drugs dispensed and procedures performed during hospital stays, coded according to the common classification of medical procedures—CCAM, and discharge diagnoses coded according to the International Statistical Classification of Diseases and Related Health Problems, Tenth Revision, ICD-10) reimbursement information since 2006. The SNDS also contains sociodemographic information on sex, age, place of residence, vital status, and complementary universal health insurance (CSS – Complémentaire santé solidaire, a system providing free access to healthcare for people with an annual income below 50% of the poverty threshold) and quintiles of deprivation index (coded from 1 to 5, 1 being the least deprived). Patients’ status for 100% reimbursement of care related to a severe and costly long-term disease (LTD) is recorded and LTD diagnosis coded according to ICD-10. Due to the medico-administrative nature of this database, clinical information such as treatment indication is not systematically recorded.

The SNDS has been extensively used to conduct pharmacoepidemiological studies, especially on the use, safety, and effectiveness of health products^22–25^.

### Study periods and populations

Originator and biosimilar products names, dates of reimbursement and formulations are presented in Supplementary Table S6.

Study start date was defined by biosimilar entry date in France which differed for each of the three studied TNF-alpha inhibitors (referred later as TNF-alpha inhibitors for simplicity), infliximab (ATC L04AB02), etanercept (ATC L04AB01), adalimumab (ATC L04AB04). The time 0 of the study was thus fixed to January 27, 2015 for infliximab users, May 10, 2016 for etanercept users and October 9, 2018 for adalimumab users. Study end date was fixed to September 30, 2021.

Patients initiating or using a TNF-alpha inhibitor from year -1 of the study to the study end date were included for a global analysis of biosimilar penetration rate, whatever the age and pathology of the patients. Then, analysis was restricted to adult patients to establish underlying pathologies with more accuracy, and the study focused on two groups of patients for individual analysis: (i) adult (≥ 18 years old) patients initiating a TNF-alpha inhibitor treatment from study start date to study end date (initiators), initiation was defined as a first dispensing of a TNF-alpha inhibitor after at least one year without any other dispensing of a TNF-alpha inhibitor; (ii) adult patients under a TNF-alpha inhibitor treatment at study start date (prevalent users), defined as having had at least one delivery of TNF-alpha inhibitor within the theoretical treatment covering period (56 days for infliximab and 28 days for etanercept and adalimumab) supplemented with a buffer period of 90 days before study start date.

Index date was defined as the date of the first TNF-alpha inhibitor delivery for initiating patients (i), and as the last delivery before stud start date for prevalent users (ii).

### Patient characteristics

Several patient characteristics were analyzed. Sociodemographic characteristics (sex, age, affiliation to complementary universal health insurance, deprivation index, region of residence) were measured at the index date.

Comorbidities were assessed within five years before the index date, using ATC and ICD-10 codes shown in Supplementary Table S7^26^. History of dispensing of corticosteroids, non-steroidal anti-inflammatory drugs (NSAID), non-biological systemic drugs on the one side and biological and targeted drugs on the other side was assessed within the year before index date. ATC codes for these drugs are presented in Supplementary Table S8.

### Identification of the Underlying Pathology

The study focused on the eight pathologies the TNF-alpha inhibitors were indicated for and reimbursed in France: rheumatoid arthritis, ankylosing spondylitis, psoriatic arthritis, psoriasis, hidradenitis suppurativa, Crohn’s disease, ulcerative colitis and non-infectious uveitis (Supplementary Table S9). As the indication is not systematically recorded in the SNDS, we identified pathology based on one of the following criteria of the patient’s medical background: (a) at least one hospitalization with an ICD-10 code as principal diagnosis (PD) or related diagnosis (RD) specific for the pathology within the five years before index date; (b) at least one hospitalization whatever the principal diagnosis, with an ICD-10 code as complication or associated diagnosis specific for the pathology within the year before index date; (c) a LTD with an ICD-10 code specific for the pathology recorded within the five years before index date; (d) in addition, for psoriasis only, at least two prescriptions of topical vitamin D derivatives (ATC code D05AX) in less than a 2-year time span within the five years before index date or at least one session of phototherapy (medical procedure coded with a specific CCAM code) within the five years before index date.

ICD-10 codes, ATC codes and medical procedure codes used for pathology definition^27–31^ are specified in Supplementary Table S9.

Patients that were categorized into multiple pathologies were further analyzed in order to retain to a unique pathology, taking into account the last indicator (hospitalization start date, LTD start date, drug dispensation date or medical procedure date) recorded in the database before index date. In case of equalities, hospitalizations with a PD or RD for this pathology (criterion (a)) and LTD (criterion (c)) were preferred indicators to establish the pathology; if still equal, the predominant number of hospitalizations (PD-RD) the year before index date was used. Other cases of equalities were left in a category “Multiple pathologies”.

Patients with no identified pathology were categorized as “Undetermined” pathology. Patients treated with a molecule not reimbursed for the identified pathology (specified in Supplementary Table S9) or with multiple pathologies were excluded.

The term “Specialty” was used to gather groups of pathologies under the terms Rheumatology (rheumatoid arthritis, ankylosing spondylitis, and psoriatic arthritis), Dermatology (psoriasis and hidradenitis suppurativa), Gastroenterology (Crohn’s disease and ulcerative colitis) and Ophthalmology (non-infectious uveitis); the patient was considered to be treated by a Rheumatologist, Dermatologist, Gastroenterologist or Ophthalmologist according the corresponding specialty group of his/her pathology.

### Follow-up duration, switch and discontinuations

Patients were followed from index date to study end date, molecule switch (including other specific biological and targeted drugs, see Supplementary Table S8), treatment discontinuation (defined as a last delivery within the study period without another delivery during the following covering period supplemented with the 90 days buffer period) or death, whichever occurred first. (i) and (ii) were schematized in Supplementary Figs. 1a and b for a sake of clarity.

A switch was identified as consecutive deliveries of the same molecule but different products. Switch was categorized into three subclasses: “transition”, which related to the switch from originator product to one of its biosimilars; “retransition”, to the switch from biosimilar to originator product; “biotransition” to the switch from biosimilar to another biosimilar of the same originator product. Time to switch was calculated as the time difference between index date and the first switch.

Treatment discontinuation was defined as a last delivery within the study period without another delivery during the following treatment covering period (56 days for infliximab, 28 days for etanercept and adalimumab) supplemented with a 90 days buffer period. Discontinuation date was set to the end date of the buffer period.

### Statistical analysis

Based on the initial global population, biosimilar initiation rate, originator product prevalence rate and transition rate were computed annually and per molecule. The biosimilar initiation rate was defined as the number of biosimilar initiations divided by the total number of initiations of the molecule. Originator product prevalence rate was estimated by dividing the number of patients having at least one delivery of the originator product by the total number of patients having at least one delivery of the considered molecule. Transition rate was defined as the number of transitions divided by the number of patients eligible to the transition, i.e. the number of patients having at least one delivery of the originator product.

Patient characteristics for populations (i) and (ii) were analyzed descriptively. Qualitative variables were summarized using effectives and percentages in each category and quantitative variables by mean and standard deviation (SD) and median and interquartile range (IQR).

All extractions from the SNDS were carried out with SAS Enterprise Guide software version 9.4; analyses were done with R version 3.5.2, using multiple packages including *dplyr*, *ggplot2*, *plotly*.

### Availability of data and materials, guidelines statement and consent to participate

In accordance with data protection legislation and the French regulation, the authors cannot publicly release the data from the French National Health Data System (Système National des Données de Santé—SNDS). However, any person or structure, public or private, for-profit or non-profit, is able to access SNDS data upon authorization from the French Data Protection Office (CNIL), in order to carry out a study, research or an evaluation in the public interest (https://www.snds.gouv.fr/SNDS/Processus-d-acces-aux-donnees and https://www.indsante.fr/). EPI-PHARE has permanent regulatory access to the data from the French National Health Data System (SNDS) via its constitutive bodies ANSM and CNAM. This permanent access is given in accordance with the French Decree No. 2016–1871 of December 26, 2016 relating to the processing of personal data called the “National Health Data System”, and French law articles Art. R. 1461–13 and 14. All requests in the database were made by duly authorized people, all methods were carried out in accordance with relevant guidelines and regulations and the study was conducted in accordance with the Declaration of Helsinki. In accordance with the permanent regulatory access granted to EPI-PHARE via ANSM and CNAM, this present work did not require the approval from the French Data Protection Authority (CNIL). The study was registered on the study register of EPI-PHARE concerning studies from SNDS data. Given that data are anonymous, no informed consent to participate was required.

## Supplementary Information

Supplementary Information 1.

Supplementary Information 2.

## Data Availability

All data generated or analysed during this study are included in this published article (and its Supplementary Information files). No additional data is available as the SNDS is not a public access database.
