# Supplementary material for: TNF-alpha inhibitors biosimilar use in France: a nationwide population-based study using the French National Health Data System
Source: Sci Rep. 2022 Nov 15;12:19569. doi: 10.1038/s41598-022-24050-7 (PMC9666557; doi:10.1038/s41598-022-24050-7)
Supplement: Supplementary file 1 — Supplementary Information 1. [file 41598_2022_24050_MOESM1_ESM.pdf]

Supplementary Figures and Tables

Table of contents

Supplementary Figure 1A. Population (i): initiators from first biosimilar market entry date......2

Supplementary Figure 1B. Population (ii): prevalent users at first biosimilar market entry date......3

Supplementary Table S1. Initiators additional characteristics at inclusion and during the study follow-up, by molecule and product type......4

Supplementary Table S2. Prevalent users additional characteristics at inclusion and during the study follow-up, by molecule......5

Supplementary Table S3a. Infliximab initiators characteristics at inclusion according to their switch pattern......6

Supplementary Table S3b. Infliximab prevalent users characteristics at inclusion according to their switch pattern......7

Supplementary Table S4a. Etanercept initiators characteristics at inclusion according to their switch pattern......8

Supplementary Table S4b. Etanercept prevalent users characteristics at inclusion according to their switch pattern......9

Supplementary Table S5a. Adalimumab initiators characteristics at inclusion according to their switch pattern......10

Supplementary Table S5b. Adalimumab prevalent users characteristics at inclusion according to their switch pattern......11

Supplementary Table S6. Names, abbreviations and dates of reimbursement of infliximab, etanercept and adalimumab originator and biosimilar products in France. Source: legifrance.fr......12

Supplementary Table S7. ICD-10 and ATC codes for comorbidities identification......13

Supplementary Table S8. ATC codes for history of treatments......15

Supplementary Table S9. ICD-10, ATC and CCAM codes for pathology identification, and molecules indicated for the studied pathologies......16

Supplementary Figure 1a. Population (i): initiators from first biosimilar market entry date

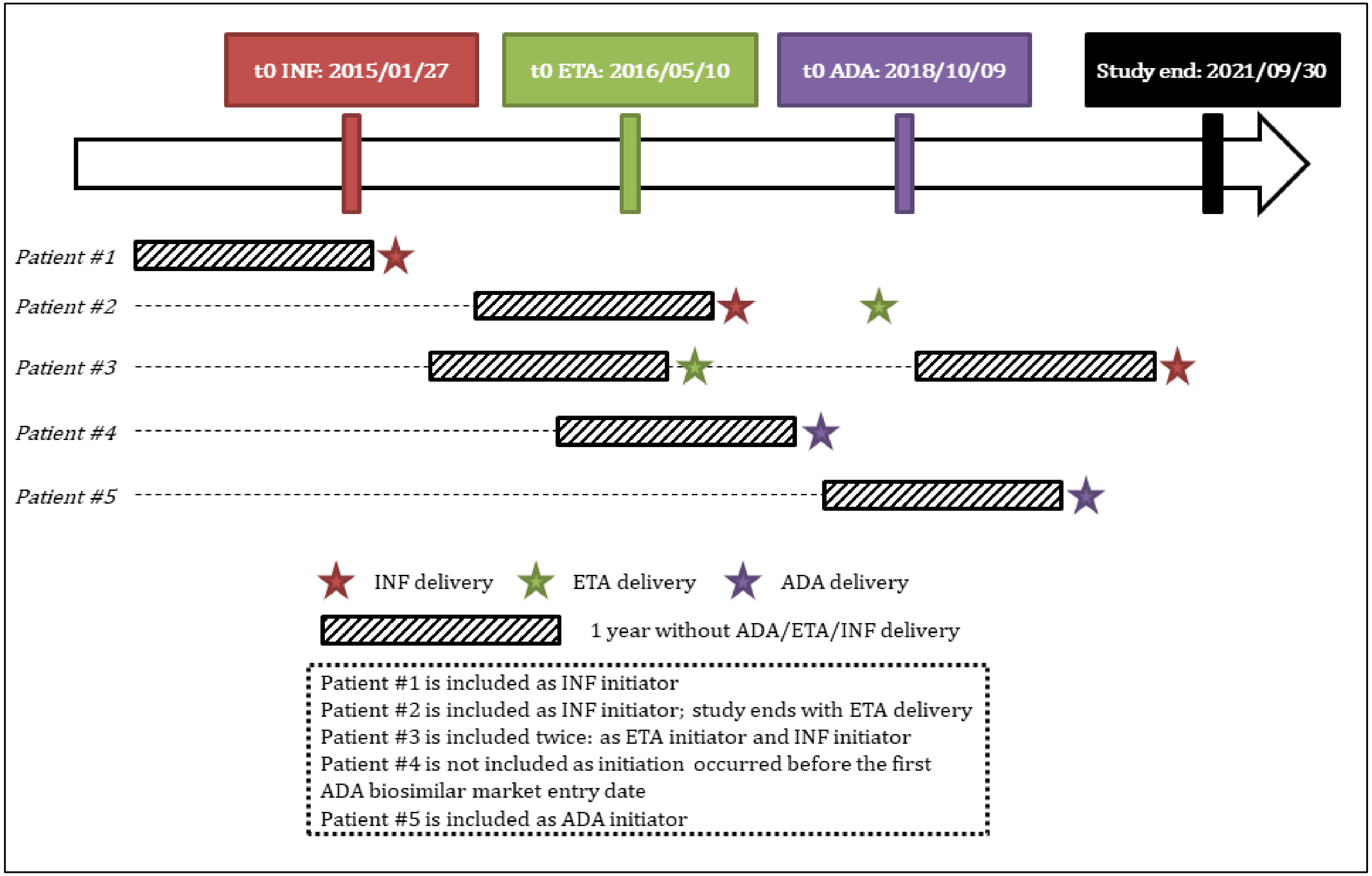

Supplementary Figure 1b. Population (ii): prevalent users at first biosimilar market entry date

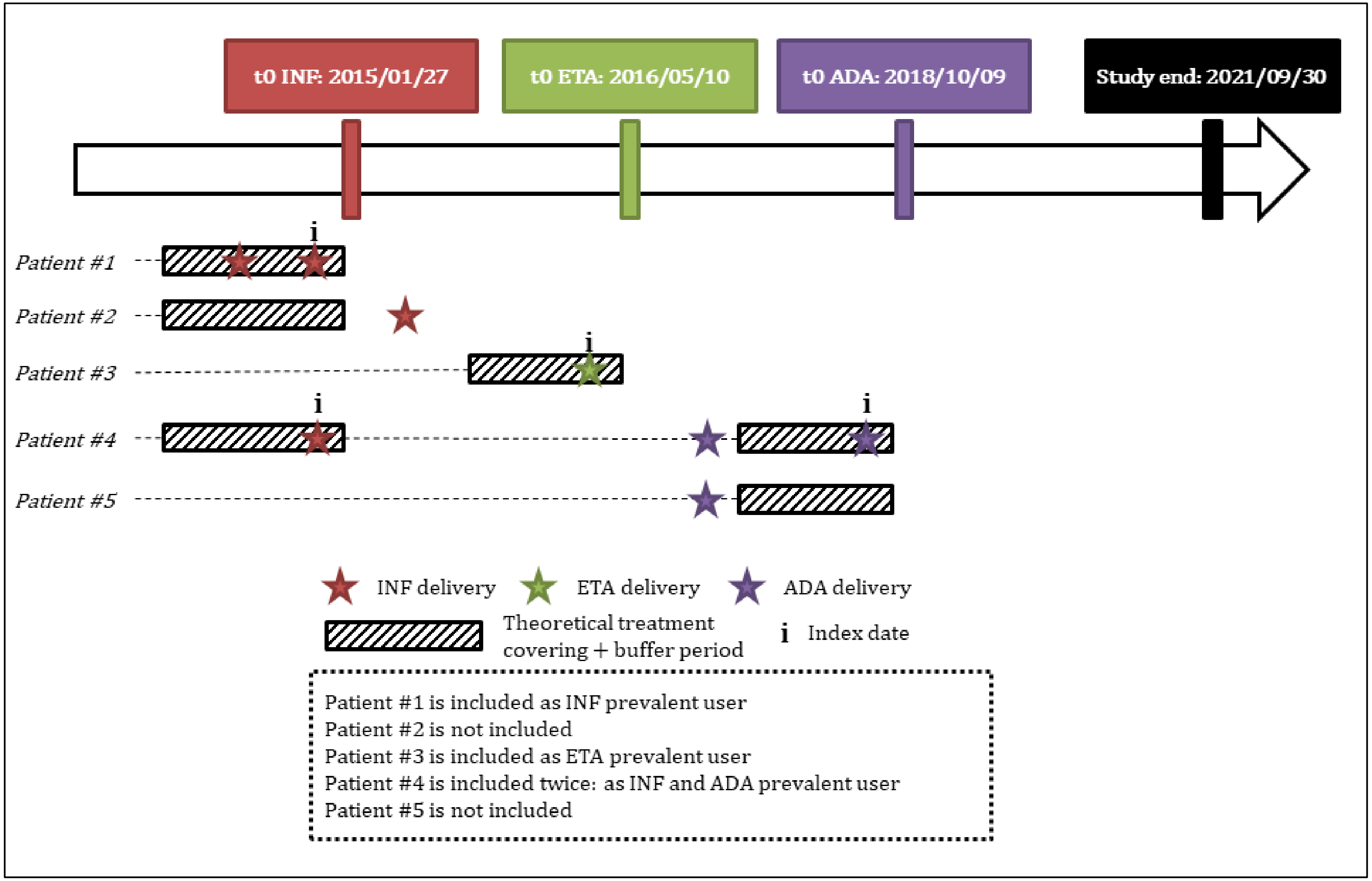

Supplementary Table S1. Initiators additional characteristics at inclusion and during the study follow-up, by molecule and product type.*

	Infliximab		Etanercept		Adalimumab	
	Biosimilar	Originator	Biosimilar	Originator	Biosimilar	Originator
**Effective**	20,208 (78)	5,742 (22)	12,713 (46)	15,014 (54)	26,033 (53)	22,735 (47)
**Year of inclusion**	-	-	-	-	-	-
0	1,139 (6)	2,643 (46)	529 (4)	5,349 (36)	5,679 (22)	9,391 (41)
1	2,721 (13)	987 (17)	1,864 (15)	3,688 (25)	8,813 (34)	6,215 (27)
2	2,874 (14)	770 (13)	2,933 (23)	2,240 (15)	11,541 (44)	7,129 (31)
3	3,198 (16)	443 (8)	2,865 (23)	1,511 (10)	-	-
4	3,347 (17)	363 (6)	3,287 (26)	1,663 (11)	-	-
5	3,441 (17)	326 (6)	1,235 (10)	563 (4)	-	-
6	3,488 (17)	210 (4)	-	-	-	-
**Deprivation index**	-	-	-	-	-	-
1 (least deprived)	3,735 (18)	864 (15)	2,154 (17)	2,837 (19)	4,645 (18)	4,103 (18)
2	3,823 (19)	980 (17)	2,563 (20)	2,977 (20)	5,228 (20)	4,458 (20)
3	4,001 (20)	1,058 (18)	2,690 (21)	2,997 (20)	5,348 (21)	4,607 (20)
4	3,891 (19)	1,191 (21)	2,567 (20)	2,825 (19)	5,021 (19)	4,413 (19)
5 (most deprived)	4,191 (21)	1,185 (21)	2,331 (18)	2,760 (18)	4,804 (18)	4,278 (19)
Missing	567 (3)	464 (8)	408 (3)	618 (4)	987 (4)	876 (4)
**CSS**	3,085 (15)	819 (14)	1,195 (9)	1,323 (9)	2,550 (10)	2,152 (9)
**History of treatment**	-	-	-	-	-	-
Corticosteroids	13,978 (69)	3,978 (69)	8,919 (70)	10,216 (68)	17,815 (68)	15,933 (70)
NSAID	7,606 (38)	2,074 (36)	8,673 (68)	10,382 (69)	14,794 (57)	9,367 (41)
Non-biological systemic drugs	10,987 (54)	3,036 (53)	7,549 (59)	7,483 (50)	13,561 (52)	11,797 (52)
Biological and targeted drugs	2,838 (14)	495 (9)	2,173 (17)	2,504 (17)	3,050 (12)	2,311 (10)
**History of frequent comorbidities**	-	-	-	-	-	-
Diabetes	1,446 (7)	451 (8)	1,077 (8)	1,210 (8)	1,308 (5)	1,123 (5)
Hypertension	1,675 (8)	565 (10)	895 (7)	1,112 (7)	910 (3)	784 (3)
Dyslipidemia	532 (3)	181 (3)	264 (2)	337 (2)	271 (1)	211 (1)
Chronic obstructive pulmonary disease	955 (5)	312 (5)	500 (4)	621 (4)	611 (2)	491 (2)
Complicated obesity	1,265 (6)	371 (6)	630 (5)	844 (6)	945 (4)	774 (3)
Heart disease	1,953 (10)	579 (10)	1,199 (9)	1,418 (9)	1,522 (6)	1,326 (6)
Hepatic insufficiency/cirrhosis	840 (4)	246 (4)	341 (3)	386 (3)	442 (2)	364 (2)
Chronic renal failure	300 (1)	109 (2)	189 (1)	200 (1)	133 (1)	138 (1)
Cancer	1,712 (8)	542 (9)	837 (7)	1,008 (7)	1,117 (4)	1,010 (4)
Smoking use disorders	1,289 (6)	353 (6)	371 (3)	442 (3)	554 (2)	550 (2)
Transplantation	157 (1)	50 (1)	96 (1)	149 (1)	31 (0)	38 (0)

* Figures are no. (%). CSS Complementary universal health insurance, Derivation index 1 refers to the least deprived quintile.

Supplementary Table S2. Prevalent users additional characteristics at inclusion and during the study follow-up, by molecule.*

	**Infliximab**	**Etanercept**	**Adalimumab**
**Effective**	20,254	31,038	53,381
**Deprivation index**	-	-	-
1 (least deprived)	3,565 (18)	5,608 (18)	9,540 (18)
2	3,740 (18)	6,120 (20)	10,691 (20)
3	4,058 (20)	6,305 (20)	10,797 (20)
4	4,025 (20)	6,185 (20)	10,449 (20)
5 (most deprived)	4,091 (20)	5,687 (18)	10,063 (19)
Missing	775 (4)	1,133 (4)	1,841 (3)
**CSS**	1,767 (9)	1,627 (5)	3,511 (7)
**History of treatment**	-	-	-
Corticosteroids	10,890 (54)	16,890 (54)	27,760 (52)
NSAID	7,524 (37)	18,019 (58)	21,247 (40)
Non-biological systemic drugs	9,025 (45)	11,825 (38)	18,653 (35)
Biological and targeted drugs	333 (2)	763 (2)	1,543 (3)
**History of frequent comorbidities**	-	-	-
Diabetes	1,188 (6)	2,200 (7)	2,701 (5)
Hypertension	1,555 (8)	2,064 (7)	2,060 (4)
Dyslipidemia	550 (3)	473 (2)	537 (1)
Chronic obstructive pulmonary disease	792 (4)	870 (3)	1,124 (2)
Complicated obesity	1,314 (6)	1,034 (3)	1,478 (3)
Heart disease	1,477 (7)	2,845 (9)	3,495 (7)
Hepatic insufficiency/cirrhosis	658 (3)	548 (2)	911 (2)
Chronic renal failure	218 (1)	293 (1)	330 (1)
Cancer	1,114 (6)	1,400 (5)	2,210 (4)
Smoking use disorders	871 (4)	431 (1)	915 (2)
Transplantation	37 (0)	53 (0)	79 (0)

* Figures are no. (%). CSS Complementary universal health insurance, Derivation index 1 refers to the least deprived quintile.

Supplementary Table S3a. Infliximab initiators characteristics at inclusion according to their switch pattern.*

	Originator only (n = 3,711)	Biosimilar only (n = 16,859)	Transition (n = 2,031)	Retransition (n = 1,173)	Biotransition (n = 2,176)
**Year of inclusion**	-	-	-	-	-
0	1,684 (45)	833 (5)	959 (47)	207 (18)	99 (5)
1	634 (17)	2,055 (12)	353 (17)	316 (27)	350 (16)
2	449 (12)	2,227 (13)	321 (16)	207 (18)	440 (20)
3	302 (8)	2,612 (15)	141 (7)	174 (15)	412 (19)
4	242 (7)	2,781 (16)	121 (6)	124 (11)	442 (20)
5	241 (6)	3,050 (18)	85 (4)	108 (9)	283 (13)
6	159 (4)	3,301 (20)	51 (3)	37 (3)	150 (7)
**Female**	1,975 (53)	8,703 (52)	1,037 (51)	589 (50)	1,085 (50)
**Age at inclusion (years)**	-	-	-	-	-
Mean (SD)	45 (16.3)	44.3 (16.3)	41.9 (15.4)	42.5 (15.9)	41.9 (15.8)
18-29	777 (21)	3,787 (22)	538 (26)	301 (26)	578 (27)
30-39	747 (20)	3,511 (21)	441 (22)	259 (22)	515 (24)
40-49	776 (21)	3,283 (19)	409 (20)	225 (19)	412 (19)
50-59	631 (17)	2,965 (18)	351 (17)	191 (16)	308 (14)
60-69	470 (13)	2,031 (12)	201 (10)	133 (11)	240 (11)
70+	310 (8)	1,282 (8)	91 (4)	64 (5)	123 (6)
**Deprivation index**	-	-	-	-	-
1 (least deprived)	539 (15)	3,135 (19)	325 (16)	187 (16)	413 (19)
2	647 (17)	3,255 (19)	333 (16)	197 (17)	371 (17)
3	679 (18)	3,345 (20)	379 (19)	235 (20)	421 (19)
4	768 (21)	3,241 (19)	423 (21)	241 (21)	409 (19)
5 (most deprived)	761 (21)	3,462 (21)	424 (21)	228 (19)	501 (23)
Missing	317 (9)	421 (2)	147 (7)	85 (7)	61 (3)
**CSS**	530 (14)	2,588 (15)	289 (14)	188 (16)	309 (14)
**Pathology**	-	-	-	-	-
**Gastro-enterology**					
Crohn's Disease	1,412 (38)	6,420 (38)	995 (49)	464 (40)	1,030 (47)
Ulcerative colitis	688 (19)	3,609 (21)	370 (18)	236 (20)	513 (24)
**Rheumatology**					
Rheumatoid arthritis	317 (9)	1,419 (8)	129 (6)	67 (6)	121 (6)
Ankylosing spondylitis	500 (13)	2,847 (17)	281 (14)	193 (16)	318 (15)
Psoriatic arthritis	93 (3)	372 (2)	49 (2)	32 (3)	44 (2)
**Dermatology**					
Psoriasis	215 (6)	752 (4)	79 (4)	51 (4)	59 (3)
**Undetermined**	486 (13)	1,440 (9)	128 (6)	130 (11)	91 (4)
**History of treatment**	-	-	-	-	-
Corticosteroids	2636 (71)	11,625 (69)	1,342 (66)	863 (74)	1,490 (68)
NSAID	1369 (37)	6,349 (38)	705 (35)	497 (42)	760 (35)
Non-biological systemic drugs	1915 (52)	8,994 (53)	1,121 (55)	650 (55)	1,343 (62)
Biological and targeted drugs	339 (9)	2,451 (15)	156 (8)	152 (13)	235 (11)
**History of frequent comorbidities**	-	-	-	-	-
Diabetes	310 (8)	1,238 (7)	141 (7)	90 (8)	118 (5)
Hypertension	393 (11)	1,403 (8)	172 (8)	117 (10)	155 (7)
Dyslipidemia	129 (3)	459 (3)	52 (3)	42 (4)	31 (1)
Chronic obstructive pulmonary disease	223 (6)	813 (5)	89 (4)	62 (5)	80 (4)
Complicated obesity	250 (7)	1042 (6)	121 (6)	100 (9)	123 (6)
Heart disease	414 (11)	1,663 (10)	165 (8)	121 (10)	169 (8)
Hepatic insufficiency/cirrhosis	175 (5)	714 (4)	71 (3)	57 (5)	69 (3)
Chronic renal failure	84 (2)	246 (1)	25 (1)	22 (2)	32 (1)
Cancer	436 (12)	1,526 (9)	106 (5)	74 (6)	112 (5)
Smoking use disorders	245 (7)	1,068 (6)	108 (5)	76 (6)	145 (7)
Transplantation	45 (1)	144 (1)	5 (0)	4 (0)	9 (0)

* Figures are no. (%) unless stated otherwise. SD standard deviation, CSS Complementary universal health insurance, Derivation index 1 refers to the least deprived quintile.

Supplementary Table S3b. Infliximab prevalent users characteristics at inclusion according to their switch pattern.*

	Originator only (n = 10,837)	Transition (n = 7,363)	Retransition (n = 2,054)
**Female**	5,825 (54)	3,451 (47)	1,030 (50)
**Age at inclusion (years)**	-	-	-
Mean (SD)	45.4 (15.6)	44.2 (14.9)	45.8 (15.1)
18-29	1,990 (18)	1,417 (19)	346 (17)
30-39	2,195 (20)	1,564 (21)	390 (19)
40-49	2,427 (22)	1,724 (23)	483 (24)
50-59	2,043 (19)	1,414 (19)	417 (20)
60-69	1,410 (13)	867 (12)	292 (14)
70+	772 (7)	377 (5)	126 (6)
**Deprivation index**	-	-	-
1 (least deprived)	1,765 (16)	1,462 (20)	338 (16)
2	1,978 (18)	1,409 (19)	353 (17)
3	2,209 (20)	1,412 (19)	437 (21)
4	2,145 (20)	1,437 (20)	443 (22)
5 (most deprived)	2,259 (21)	1,441 (20)	391 (19)
Missing	481 (4)	202 (3)	92 (4)
**CSS**	1,009 (9)	603 (8)	155 (8)
**Pathology**	-	-	-
**Gastro-enterology**	-	-	-
Crohn's Disease	4,258 (39)	3,512 (48)	737 (36)
Ulcerative colitis	1,600 (15)	971 (13)	213 (10)
**Rheumatology**	-	-	-
Rheumatoid arthritis	1,143 (11)	666 (9)	223 (11)
Ankylosing spondylitis	2,550 (24)	1,631 (22)	644 (31)
Psoriatic arthritis	431 (4)	212 (3)	90 (4)
**Dermatology**	-	-	-
Psoriasis	656 (6)	306 (4)	114 (6)
**Undetermined**	199 (2)	65 (1)	33 (2)
**History of treatment**	-	-	-
Corticosteroids	6,318 (58)	3,541 (48)	1,031 (50)
NSAID	4,226 (39)	2,462 (33)	836 (41)
Non-biological systemic drugs	4,984 (46)	3,194 (43)	847 (41)
Biological and targeted drugs	238 (2)	59 (1)	36 (2)
**History of frequent comorbidities**	-	-	-
Diabetes	700 (6)	358 (5)	130 (6)
Hypertension	914 (8)	491 (7)	150 (7)
Dyslipidemia	326 (3)	169 (2)	55 (3)
Chronic obstructive pulmonary disease	456 (4)	262 (4)	74 (4)
Complicated obesity	731 (7)	440 (6)	143 (7)
Heart disease	904 (8)	420 (6)	153 (7)
Hepatic insufficiency/cirrhosis	375 (3)	207 (3)	76 (4)
Chronic renal failure	134 (1)	66 (1)	18 (1)
Cancer	660 (6)	352 (5)	102 (5)
Smoking use disorders	507 (5)	279 (4)	85 (4)
Transplantation	30 (0)	7 (0)	0 (0)

* Figures are no. (%) unless stated otherwise. SD standard deviation, CSS Complementary universal health insurance, Derivation index 1 refers to the least deprived quintile.

Supplementary Table S4a. Etanercept initiators characteristics at inclusion according to their switch pattern.*

	**Originator only** (n = 13,439)	**Biosimilar only** (n = 11,732)	**Transition** (n = 1,575)	**Retransition** (n = 692)	**Biotransition** (n = 289)
**Year of inclusion**	-	-	-	-	-
0	4,636 (34)	481 (4)	713 (45)	46 (7)	2 (1)
1	3,245 (24)	1,688 (14)	443 (28)	141 (20)	35 (12)
2	1,992 (15)	2,679 (23)	248 (16)	178 (26)	76 (26)
3	1,406 (10)	2,606 (22)	105 (7)	163 (24)	96 (33)
4	1,603 (12)	3,065 (26)	60 (4)	150 (22)	72 (25)
5	557 (4)	1,213 (10)	6 (0)	14 (2)	8 (3)
**Female**	8,612 (64)	7,249 (62)	917 (58)	454 (66)	204 (71)
**Age at inclusion (years)**	-	-	-	-	-
Mean (SD)	50.6 (15.1)	52 (15.1)	50.9 (15.3)	52 (15.6)	50 (14.8)
18-29	1,159 (9)	921 (8)	139 (9)	56 (8)	27 (9)
30-39	2,255 (17)	1,837 (16)	276 (18)	109 (16)	46 (16)
40-49	2,997 (22)	2,255 (19)	314 (20)	140 (20)	71 (25)
50-59	3,187 (24)	2,910 (25)	390 (25)	170 (25)	70 (24)
60-69	2,267 (17)	2,180 (19)	264 (17)	107 (15)	41 (14)
70+	1,574 (12)	1,629 (14)	192 (12)	110 (16)	34 (12)
**Deprivation index**	-	-	-	-	-
1 (least deprived)	2,519 (19)	1,979 (17)	318 (20)	130 (19)	45 (16)
2	2,683 (20)	2,358 (20)	294 (19)	150 (22)	55 (19)
3	2,718 (20)	2,476 (21)	279 (18)	144 (21)	70 (24)
4	2,505 (19)	2,381 (20)	320 (20)	129 (19)	57 (20)
5 (most deprived)	2,434 (18)	2,163 (18)	326 (21)	117 (17)	51 (18)
Missing	580 (4)	375 (3)	38 (2)	22 (3)	11 (4)
**CSS**	1,217 (9)	1,110 (9)	106 (7)	70 (10)	15 (5)
**Pathology**	-	-	-	-	-
**Rheumatology**	-	-	-	-	-
Rheumatoid arthritis	4,929 (37)	5,525 (47)	689 (44)	321 (46)	151 (52)
Ankylosing spondylitis	4,810 (36)	4,118 (35)	546 (35)	232 (34)	91 (31)
Psoriatic arthritis	796 (6)	635 (5)	124 (8)	37 (5)	16 (6)
**Dermatology**	-	-	-	-	-
Psoriasis	1,232 (9)	517 (4)	88 (6)	42 (6)	14 (5)
**Undetermined**	1,672 (12)	937 (8)	128 (8)	60 (9)	17 (6)
**History of treatment**	-	-	-	-	-
Corticosteroids	9,160 (68)	8,206 (70)	1,056 (67)	499 (72)	214 (74)
NSAID	9,289 (69)	7,996 (68)	1,093 (69)	471 (68)	206 (71)
Non-biological systemic drugs	6,592 (49)	6,976 (59)	891 (57)	374 (54)	199 (69)
Biological and targeted drugs	2,292 (17)	1,977 (17)	212 (13)	137 (20)	59 (20)
**History of frequent comorbidities**	-	-	-	-	-
Diabetes	1,085 (8)	977 (8)	125 (8)	76 (11)	24 (8)
Hypertension	988 (7)	813 (7)	124 (8)	63 (9)	19 (7)
Dyslipidemia	288 (2)	238 (2)	49 (3)	22 (3)	4 (1)
Chronic obstructive pulmonary disease	549 (4)	449 (4)	72 (5)	39 (6)	12 (4)
Complicated obesity	749 (6)	582 (5)	95 (6)	35 (5)	13 (4)
Heart disease	1,272 (9)	1,097 (9)	146 (9)	79 (11)	23 (8)
Hepatic insufficiency/cirrhosis	347 (3)	304 (3)	39 (2)	33 (5)	4 (1)
Chronic renal failure	168 (1)	177 (2)	32 (2)	11 (2)	1 (0)
Cancer	897 (7)	761 (6)	111 (7)	54 (8)	22 (8)
Smoking use disorders	379 (3)	330 (3)	63 (4)	33 (5)	8 (3)
Transplantation	137 (1)	88 (1)	12 (1)	8 (1)	0 (0)

* Figures are no. (%) unless stated otherwise. SD standard deviation, CSS Complementary universal health insurance, Derivation index 1 refers to the least deprived quintile.

Supplementary Table S4b. Etanercept prevalent users characteristics at inclusion according to their switch pattern.*

	Originator only (n = 25,131)	Transition (n = 3,724)	Retransition (n = 2,183)
**Female**	14,263 (57)	1,917 (51)	1,183 (54)
**Age at inclusion (years)**	-	-	-
Mean (SD)	53.2 (14.6)	53.2 (14.1)	54.2 (14.4)
18-29	1,413 (6)	203 (5)	100 (5)
30-39	3,266 (13)	459 (12)	259 (12)
40-49	5,430 (22)	779 (21)	456 (21)
50-59	6,261 (25)	961 (26)	549 (25)
60-69	5,387 (21)	858 (23)	480 (22)
70+	3,374 (13)	464 (12)	339 (16)
**Deprivation index**	-	-	-
1 (least deprived)	4,503 (18)	684 (18)	421 (19)
2	4,969 (20)	730 (20)	421 (19)
3	5,046 (20)	802 (22)	457 (21)
4	5,001 (20)	746 (20)	438 (20)
5 (most deprived)	4,621 (18)	670 (18)	396 (18)
Missing	991 (4)	92 (2)	50 (2)
**CSS**	1,393 (6)	146 (4)	88 (4)
**Pathology**	-	-	-
**Rheumatology**	-	-	-
Rheumatoid arthritis	10,197 (41)	1,630 (44)	995 (46)
Ankylosing spondylitis	8,991 (36)	1,424 (38)	776 (36)
Psoriatic arthritis	1,812 (7)	268 (7)	178 (8)
**Dermatology**	-	-	-
Psoriasis	2,891 (12)	248 (7)	143 (7)
**Undetermined**	1,240 (5)	154 (4)	91 (4)
**History of treatment**	-	-	-
Corticosteroids	14,058 (56)	1,729 (46)	1,103 (51)
NSAID	14,775 (59)	2,003 (54)	1,241 (57)
Non-biological systemic drugs	9,474 (38)	1,525 (41)	826 (38)
Biological and targeted drugs	695 (3)	47 (1)	21 (1)
**History of frequent comorbidities**	-	-	-
Diabetes	1,832 (7)	228 (6)	140 (6)
Hypertension	1,693 (7)	213 (6)	158 (7)
Dyslipidemia	384 (2)	64 (2)	25 (1)
Chronic obstructive pulmonary disease	736 (3)	75 (2)	59 (3)
Complicated obesity	857 (3)	109 (3)	68 (3)
Heart disease	2,320 (9)	314 (8)	211 (10)
Hepatic insufficiency/cirrhosis	454 (2)	61 (2)	33 (2)
Chronic renal failure	243 (1)	32 (1)	18 (1)
Cancer	1,165 (5)	142 (4)	93 (4)
Smoking use disorders	356 (1)	48 (1)	27 (1)
Transplantation	47 (0)	4 (0)	2 (0)

* Figures are no. (%) unless stated otherwise. SD standard deviation, CSS Complementary universal health insurance, Derivation index 1 refers to the least deprived quintile.

Supplementary Table S5a. Adalimumab initiators characteristics at inclusion according to their switch pattern.*

	Originator only (n = 21,339)	Biosimilar only (n = 23,812)	Transition (n = 1,396)	Retransition (n = 1,592)	Biotransition (n = 629)
**Year of inclusion**	-	-	-	-	-
0	8,533 (40)	4,922 (21)	858 (61)	551 (35)	206 (33)
1	5,884 (28)	7,861 (33)	331 (24)	691 (43)	261 (41)
2	6,922 (32)	11,029 (46)	207 (15)	350 (22)	162 (26)
**Female**	11,881 (56)	13,102 (55)	718 (51)	884 (56)	393 (62)
**Age at inclusion (years)**	-	-	-	-	-
Mean (SD)	43.7 (15.4)	45.4 (15)	42.9 (15.6)	42.1 (15.2)	43.2 (14.8)
18-29	4,540 (21)	4,065 (17)	322 (23)	385 (24)	126 (20)
30-39	4,742 (22)	5,034 (21)	321 (23)	375 (24)	146 (23)
40-49	4,549 (21)	5,147 (22)	279 (20)	339 (21)	146 (23)
50-59	3,870 (18)	5,040 (21)	240 (17)	257 (16)	115 (18)
60-69	2,363 (11)	3,009 (13)	153 (11)	156 (10)	66 (10)
70+	1,275 (6)	1,517 (6)	81 (6)	80 (5)	30 (5)
**Deprivation index**	-	-	-	-	-
1 (least deprived)	3,836 (18)	4,215 (18)	267 (19)	316 (20)	114 (18)
2	4,201 (20)	4,763 (20)	257 (18)	324 (20)	141 (22)
3	4,354 (20)	4,941 (21)	253 (18)	299 (19)	108 (17)
4	4,124 (19)	4,595 (19)	289 (21)	304 (19)	122 (19)
5 (most deprived)	4,016 (19)	4,377 (18)	262 (19)	300 (19)	127 (20)
Missing	808 (4)	921 (4)	68 (5)	49 (3)	17 (3)
**CSS**	2,013 (9)	2,309 (10)	139 (10)	175 (11)	66 (10)
**Pathology**	-	-	-	-	-
**Gastro-enterology**	-	-	-	-	-
Crohn's Disease	6,643 (31)	4,808 (20)	484 (35)	605 (38)	170 (27)
Ulcerative colitis	3,957 (19)	2,361 (10)	165 (12)	267 (17)	69 (11)
**Rheumatology**	-	-	-	-	-
Rheumatoid arthritis	1,622 (8)	3,665 (15)	95 (7)	128 (8)	95 (15)
Ankylosing spondylitis	3,498 (16)	7,946 (33)	313 (22)	306 (19)	197 (31)
Psoriatic arthritis	620 (3)	1,308 (5)	44 (3)	49 (3)	30 (5)
**Dermatology**	-	-	-	-	-
Psoriasis	1,865 (9)	1,786 (8)	148 (11)	108 (7)	32 (5)
Hidradenitis Suppurativa	252 (1)	43 (0)	15 (1)	8 (1)	1 (0)
**Ophthalmology**	-	-	-	-	-
Uveitis	511 (2)	166 (1)	27 (2)	35 (2)	3 (0)
**Undetermined**	2,371 (11)	1,729 (7)	105 (8)	86 (5)	32 (5)
**History of treatment**	-	-	-	-	-
Corticosteroids	14,973 (70)	16,227 (68)	960 (69)	1,150 (72)	438 (70)
NSAID	8,723 (41)	13,733 (58)	644 (46)	717 (45)	344 (55)
Non-biological systemic drugs	11,069 (52)	12,348 (52)	728 (52)	858 (54)	355 (56)
Biological and targeted drugs	2,173 (10)	2,835 (12)	138 (10)	132 (8)	83 (13)
**History of frequent comorbidities**	-	-	-	-	-
Diabetes	1,053 (5)	1,204 (5)	70 (5)	75 (5)	29 (5)
Hypertension	720 (3)	805 (3)	64 (5)	76 (5)	29 (5)
Dyslipidemia	192 (1)	246 (1)	19 (1)	21 (1)	4 (1)
Chronic obstructive pulmonary disease	469 (2)	539 (2)	22 (2)	56 (4)	16 (3)
Complicated obesity	717 (3)	848 (4)	57 (4)	68 (4)	29 (5)
Heart disease	1,232 (6)	1,379 (6)	94 (7)	98 (6)	45 (7)
Hepatic insufficiency/cirrhosis	343 (2)	394 (2)	21 (2)	41 (3)	7 (1)
Chronic renal failure	130 (1)	120 (1)	8 (1)	10 (1)	3 (0)
Cancer	939 (4)	1,024 (4)	71 (5)	67 (4)	26 (4)
Smoking use disorders	510 (2)	477 (2)	40 (3)	57 (4)	20 (3)
Transplantation	34 (0)	29 (0)	4 (0)	2 (0)	0 (0)

* Figures are no. (%) unless stated otherwise. SD standard deviation, CSS Complementary universal health insurance, Derivation index 1 refers to the least deprived quintile.

Supplementary Table S5b. Adalimumab prevalent users characteristics at inclusion according to their switch pattern.*

	Originator only (n = 44,096)	Transition (n = 6,230)	Retransition (n = 3,055)
**Female**	22,473 (51)	2,743 (44)	1522 (50)
**Age at inclusion (years)**	-	-	-
Mean (SD)	46.4 (15.3)	47.9 (14.8)	47.5 (15)
18-29	6,871 (16)	752 (12)	416 (14)
30-39	8,883 (20)	1,191 (19)	580 (19)
40-49	9,854 (22)	1,440 (23)	701 (23)
50-59	9,096 (21)	1,367 (22)	657 (22)
60-69	6,034 (14)	968 (16)	457 (15)
70+	3,358 (8)	512 (8)	244 (8)
**Deprivation index**	-	-	-
1 (least deprived)	7,781 (18)	1,219 (20)	540 (18)
2	8,792 (20)	1,260 (20)	639 (21)
3	8,960 (20)	1,212 (19)	625 (20)
4	8,590 (19)	1,260 (20)	599 (20)
5 (most deprived)	8,373 (19)	1,118 (18)	572 (19)
Missing	1,600 (4)	161 (3)	80 (3)
**CSS**	3,036 (7)	309 (5)	166 (5)
**Pathology**	-	-	-
**Gastro-enterology**	-	-	-
Crohn's Disease	14,801 (34)	1,628 (26)	792 (26)
Ulcerative colitis	4,467 (10)	358 (6)	183 (6)
**Rheumatology**	-	-	-
Rheumatoid arthritis	5,828 (13)	1,058 (17)	480 (16)
Ankylosing spondylitis	10,193 (23)	1,984 (32)	1,070 (35)
Psoriatic arthritis	1,936 (4)	343 (6)	154 (5)
**Dermatology**	-	-	-
Psoriasis	4,258 (10)	570 (9)	256 (8)
Hidradenitis Suppurativa	184 (0)	14 (0)	12 (0)
**Ophthalmology**	-	-	-
Uveitis	486 (1)	31 (0)	19 (1)
**Undetermined**	1,943 (4)	244 (4)	89 (3)
**History of treatment**	-	-	-
Corticosteroids	23,492 (53)	2,828 (45)	1,440 (47)
NSAID	17,343 (39)	2,569 (41)	1,335 (44)
Non-biological systemic drugs	15,456 (35)	2,206 (35)	991 (32)
Biological and targeted drugs	1,367 (3)	119 (2)	57 (2)
**History of frequent comorbidities**	-	-	-
Diabetes	2,248 (5)	293 (5)	160 (5)
Hypertension	1,738 (4)	197 (3)	125 (4)
Dyslipidemia	463 (1)	47 (1)	27 (1)
Chronic obstructive pulmonary disease	968 (2)	107 (2)	49 (2)
Complicated obesity	1,260 (3)	141 (2)	77 (3)
Heart disease	2,919 (7)	380 (6)	196 (6)
Hepatic insufficiency/cirrhosis	761 (2)	110 (2)	40 (1)
Chronic renal failure	282 (1)	29 (0)	19 (1)
Cancer	1,896 (4)	205 (3)	109 (4)
Smoking use disorders	779 (2)	79 (1)	57 (2)
Transplantation	64 (0)	11 (0)	4 (0)

* Figures are no. (%) unless stated otherwise. SD standard deviation, CSS Complementary universal health insurance, Derivation index 1 refers to the least deprived quintile.

Supplementary Table S6. Names, abbreviations,dates of reimbursement and formulations of infliximab, etanercept and adalimumab originator and biosimilar products in France. Sources: legifrance.fr, base-donnees-publique.medicaments.gouv.fr

Molecule	Product brand name	Abbreviation / Clinical trial name	Date of reimbursement	Formulation
infliximab	Remicade®	Originator	12/03/2000	- 100mg, powder for infusion
infliximab	Remsima®	CT-P13	27/01/2015	- 100mg, powder for infusion<br>- 120mg, prefilled syringe<br>- 120mg prefilled pen
infliximab	Inflectra®	CT-P13	27/01/2015	- 100mg, powder for infusion
infliximab	Flixabi®	SB2	18/10/2016	- 100mg, powder for infusion
infliximab	Zessly®	PF-06438179/GP1111	21/02/2019	- 100mg, powder for infusion
etanercept	Enbrel®	Originator	05/09/2003	- 10mg, powder for infusion<br>- 25mg, powder for infusion<br>- 25mg, prefilled syringe<br>- 25mg, prefilled pen<br>- 50mg, prefilled syringe<br>- 50mg, prefilled pen
etanercept	Benepali®	SB4	10/05/2016	- 25mg, prefilled syringe<br>- 25mg, prefilled pen<br>- 50mg, prefilled syringe
etanercept	Erelzi®	GP2015	24/11/2017	- 25mg, prefilled syringe<br>- 25mg, prefilled pen<br>- 50mg, prefilled syringe
etanercept	Nepexto®	YLB113	21/05/2021	- 25mg, prefilled syringe<br>- 25mg, prefilled pen<br>- 50mg, prefilled syringe
adalimumab	Humira®	Originator	09/03/2005	- 20mg, prefilled syringe<br>- 40mg, prefilled pen<br>- 40mg, prefilled syringe<br>- 40mg, infusion solution<br>- 80mg, prefilled pen<br>- 80mg, prefilled syringe
adalimumab	Amgevita®	ABP 501	09/10/2018	- 20mg, prefilled syringe<br>- 40mg, prefilled pen<br>- 40mg, prefilled syringe
adalimumab	Imraldi®	SB5	17/10/2018	- 40mg, prefilled pen<br>- 40mg, prefilled syringe
adalimumab	Hyrimoz®	GP2017	30/10/2018	- 40mg, prefilled pen<br>- 40mg, prefilled syringe
adalimumab	Hulio®	FKB327	20/12/2018	- 20mg, prefilled syringe<br>- 40mg, prefilled pen<br>- 40mg, prefilled syringe<br>- 40mg, infusion solution
adalimumab	Idacio®	MSB11022	20/08/2019	- 40mg, prefilled pen<br>- 40mg, prefilled syringe<br>- 40mg, infusion solution
adalimumab	Yuflyma®	CT-P17	31/01/2021	- 40mg, prefilled pen<br>- 40mg, prefilled syringe<br>- 40mg, infusion solution<br>- 80mg, prefilled pen
adalimumab	Amsparity®	PF-06410293	30/04/2021	- 40mg, prefilled pen<br>- 40mg, prefilled syringe

Supplementary Table S7. ICD-10 and ATC codes for comorbidities identification

Comorbidity	ICD-10 codes for hospitalization and LTD	ATC Codes (at least 3 dispensings within the year)
Diabetes	E10, E11, E12, E13, E14	A10AB01 A10AB03 A10AB04 A10AB05 A10AB06 A10AC01 A10AC03 A10AC04 A10AD01 A10AD03 A10AD04 A10AD05 A10AE01 A10AE02 A10AE03 A10AE04 A10AE05 A10AE30 A10BA02 A10BB01 A10BB03 A10BB04 A10BB06 A10BB07 A10BB09 A10BB12 A10BD02 A10BD03 A10BD05 A10BD07 A10BD08 A10BD10 A10BD15 A10BD16 A10BF01 A10BF02 A10BG02 A10BG03 A10BH01 A10BH02 A10BH03 A10BX02 A10BX04 A10BX07 A10BX09 A10BX10 A10BX11 A10BX12
Hypertension	I10	C02AB02 C02AC01 C02AC02 C02AC05 C02AC06 C02CA01 C02CA02 C02CA06 C02DC01 C02LA01 C03AA01 C03AA03 C03BA04 C03BA10 C03BA11 C03BX03 C03CA01 C03CA02 C03CA03 C03DA01 C03DA02 C03DA04 C03DB01 C03EA01 C03EA04 C03EB01 C07AA02 C07AA03 C07AA05 C07AA06 C07AA12 C07AA15 C07AA16 C07AA23 C07AB02 C07AB03 C07AB04 C07AB05 C07AB07 C07AB08 C07AB12 C07AG01 C07AG02 C07BA02 C07BB02 C07BB03 C07BB07 C07BB12 C07CA03 C07DA06 C07FB02 C07FB03 C08CA01 C08CA02 C08CA03 C08CA04 C08CA05 C08CA06 C08CA09 C08CA11 C08CA13 C08CX01 C08DA01 C08DB01 C08EA02 C09AA01 C09AA02 C09AA03 C09AA04 C09AA05 C09AA06 C09AA07 C09AA08 C09AA09 C09AA10 C09AA13 C09AA15 C09AA16 C09BA01 C09BA02 C09BA03 C09BA04 C09BA05 C09BA07 C09BA09 C09BA15 C09BB02 C09BB04 C09BB10 C09CA01 C09CA02 C09CA03 C09CA04 C09CA06 C09CA07 C09CA08 C09DA01 C09DA02 C09DA03 C09DA04 C09DA06 C09DA07 C09DA0 C09DB01 C09DB02 C09DB04 C09XA02 C09XA52 C10BX03
Dyslipidemia	E78	C10AA01 C10AA03 C10AA05 C10AA07 C10BA02 C10BX02 C10BX03 C10AB01 C10AB02 C10AB04 C10AB05 C10AB08 C10AC01 C10AD02 C10AX02 C10AX03 C10AX06 C10AX09
COPD	J40 J41 J42 J43 J44 J45 J46 J47 J961 J969 J98	
Complicated obesity	E65 E66 E67 E68	A08A V06A
Heart disease		
Cardiac failure	I50 J81 I11 I13 K761	
Ischemic disease	I20 I21 I22 I23 I24 I25	
Atrial fibrillation	I48	
Valvular disease	I05 I06 I07 I08 I34 I35 I36 I37 I38 I39	
Cardiac rhythm disorder	I44 I45 I47 I48 I49	
Obliterating arteritis of the lower limbs	I702 I739 I740 I743 I744 I745	

Stroke	G46 I60 I61 I62 I63 I64 I65 I66 I67 I68 I69 G45	
Chronic renal failure	N18	
Cancer	All CIM10 "C" codes CIM10 codes from D00 to D09 D37-D48 Z510 Z511	
Hepatic insufficiency/cirrhosis	B18 I85 K70 K71 K72 K73 K74 K75 K76 Z944	
Transplantation	Z94 T86	
Smoking use disorders	Z716 F17 T652 Z720	N06AX12 N07BA

Supplementary Table S8. ATC codes for history of treatments

Therapeutic class	ATC codes
Corticosteroids	budesonide (A07EA06)<br>dexamethasone (S01BA01) prednisolone (S01BA04) fluorometholone (S01BA07) medrysone (S01BA08) rimexolone (S01BA13) fluocinolone acetonide (S01BA15)<br>methylprednisolone (H02AB04) prednisolone (H02AB06) prednisone (H02AB07)<br>hydrocortisone (D07AA02) hydrocortisone butyrate (D07AB02) desonide (D07AB08) triamcinolone (D07AB09) betamethasone (D07AC01) fluclorolone (D07AC02) desoximetasone (D07AC03) fluorocinolone acetonide (D07AC04) fluocortolone (D07AC05) diflucortolone (D07AC06) fluocinonide (D07AC08) amcinonide (D07AC11) hydrocortisone aceponate (D07AC16) fluticasone (D07AC17) difluprednate (D07AC19) clobetasol (D07AD01) halcinonide (D07AD02)
Nonsteroidal anti-inflammatory drug	ndometacin (M01AB01) diclofenac (M01AB05) etodolac (M01AB08) aceclofenac (M01AB16) piroxicam (M01AC01) tenoxicam (M01AC02) meloxicam (M01AC06)<br>ibuprofen (M01AE01) naproxen (M01AE02) ketoprofen (M01AE03) flurbiprofen (M01AE09) tiaprofenic acid (M01AE11) alminoprofen (M01AE16)<br>celecoxib (M01AH01) etoricoxib (M01AH05)<br>nabumetone (M01AX01) niflumic acid (M01AX02) nimesulide (M01AX17) diacerein (M01AX21) morniflumate (M01AX22)
Non-biological systemic drugs	sulfasalazine (A07EC01) mesalazine (A07EC02) olsalazine (A07EC03)<br>acitretine (D05BB02)<br>tacrolimus (D11AH01)<br>mycophenolic acid (L04AA06) leflunomide (L04AA13)<br>ciclosporine (L04AD01)<br>azathioprine (L04AX01) methotrexate (L04AX03)
Biological and targeted drugs	abatacept (L04AA24) apremilast (L04AA32) vedolizumab (L04AA33)<br>tofacitinib (L04AA29) baricitinib (L04AA37) upadacitinib (L04AA44) filgotinib (L04AA45)<br>cerolizumab pegol (L04AB05) golimumab (L04AB06)<br>anakinra (L04AC03) ustekinumab (L04AC05) tocilizumab (L04AC07) secukinumab (L04AC10) brodalumab (L04AC12) ixekizumab (L04AC13) sarilumab (L04AC14) guselkumab (L04AC16) tidrakizumab (L04AC17) risankizumab (L04AC18)<br>rituximab (L01XC02)

Supplementary Table S9. ICD-10, ATC and CCAM codes for pathology identification, and molecules indicated for the studied pathologies.

**Specialty**	**Pathology**	**ICD-10 codes for Long term disease or hospitalization**	**ATC codes or medical procedures codes (CCAM)**	**Reimbursed anti-TNF-alpha molecules**
Rheumatology	Rheumatoid arthritis	M05<br>M06 except M06.1 and M06.4		Infliximab, Etanercept, Adalimumab
	Psoriatic arthritis	M07 except M07.4 and M07.5		Infliximab, Etanercept, Adalimumab
	Ankylosing spondylitis	M45, M46		Infliximab, Etanercept, Adalimumab
Dermatology	Psoriasis	L40	Topical vitamin D derivatives : D05AX02, D05AX03, D05AX04 D05AX05, D05AX52<br>Phototherapy : QZRP002, QZRP003	Infliximab, Etanercept, Adalimumab
	Hidradenitis suppurativa	L732		Adalimumab
Gastro-enterology	Crohn's disease	K50, M07.4		Infliximab, Adalimumab
	Ulcerative colitis	K51, M07.5		Infliximab, Adalimumab
Ophthalmology	Uveitis	H20, H22.1, H15, H30, H441		Adalimumab
