# Supplementary material for: TNF-alpha inhibitors biosimilar use in France: a nationwide population-based study using the French National Health Data System
Source: Sci Rep. 2022 Nov 15;12:19569. doi: 10.1038/s41598-022-24050-7 (PMC9666557; doi:10.1038/s41598-022-24050-7)
Supplement: Supplementary file 2 — Supplementary Information 2. [file 41598_2022_24050_MOESM2_ESM.html]

Supplementary material: Sankey diagrams showing switch pathways of TNF-alpha inhibitors initiators and prevalent users


# Supplementary material: Sankey diagrams showing switch pathways of TNF-alpha inhibitors initiators and prevalent users

- Initiators pathways
  - Sankey Diagram showing the pathway of infliximab initiating patients (>500-patient flows)
  - Sankey Diagram showing the pathway of etanercept initiating patients (>500-patient flows)
  - Sankey Diagram showing the pathway of adalimumab initiating patients (>500-patient flows)
- Prevalent users pathways
  - Sankey Diagram showing the pathway of infliximab prevalent users (>500-patient flows)
  - Sankey Diagram showing the pathway of etanercept prevalent users (>500-patient flows)
  - Sankey Diagram showing the pathway of adalimumab prevalent users (>500-patient flows)

# Initiators pathways

## Sankey Diagram showing the pathway of infliximab initiating patients (>500-patient flows)

*ORI: originator; SIM: Biosimilar; END: death or molecule change*

*Median time to transition in ORI initiators: 1.6 years*

*Median time to retransition/biotransition in SIM initiators: 0.5/1.2 years*

*Median time to study discontinuation if no switch : 0.8 years*

## Sankey Diagram showing the pathway of etanercept initiating patients (>500-patient flows)

*ORI: originator; SIM: Biosimilar; END: death or molecule change*

*Median time to transition in ORI initiators: 1.6 years*

*Median time to retransition/biotransition in SIM initiators: 0.4/0.5 years*

*Median time to study discontinuation if no switch : 0.8 years*

## Sankey Diagram showing the pathway of adalimumab initiating patients (>500-patient flows)

*ORI: originator; SIM: Biosimilar; END: death or molecule change*

*Median time to transition in ORI initiators: 0.6 years*

*Median time to retransition/biotransition in SIM initiators: 0.3/0.4 years*

*Median time to study discontinuation if no switch : 0.7 years*

# Prevalent users pathways

## Sankey Diagram showing the pathway of infliximab prevalent users (>500-patient flows)

*ORI: originator; SIM: Biosimilar; END: death or molecule change*

*Median time to transition/retransition/biotransition: 3.1/3.5/5 years*

*Median time to study discontinuation if no switch : 1.9 years*

## Sankey Diagram showing the pathway of etanercept prevalent users (>500-patient flows)

*ORI: originator; SIM: Biosimilar; END: death or molecule change*

*Median time to transition/retransition/biotransition: 2.9/3.5/3.7 years*

*Median time to study discontinuation if no switch : 3.6 years*

## Sankey Diagram showing the pathway of adalimumab prevalent users (>500-patient flows)

*ORI: originator; SIM: Biosimilar; END: death or molecule change*

*Median time to transition/retransition/biotransition: 1.1/1.5/1.7 years*

*Median time to study discontinuation if no switch : 3 years*
